# Developing core outcome sets for clinical trials: issues to consider

**DOI:** 10.1186/1745-6215-13-132

**Published:** 2012-08-06

**Authors:** Paula R Williamson, Douglas G Altman, Jane M Blazeby, Mike Clarke, Declan Devane, Elizabeth Gargon, Peter Tugwell

**Affiliations:** 1 Department of Biostatistics, University of Liverpool, Shelley’s Cottage, Brownlow Street, Liverpool,, L69 3GS, UK; 2 University of Oxford, Centre for Statistics in Medicine, Wolfson College Annexe, Linton Road, Oxford, OX2 6UD, UK; 3 University of Bristol, School of Social and Community Medicine, 39 Whatley Road, Bristol, BS8 2PS, UK; 4 Queens University Belfast, All-Ireland Hub for Trials Methodology Research, Institute of Clinical Sciences, Block B, Royal Victoria Hospital, Grosvenor Road, Belfast, BT12 6BJ, UK; 5 National University of Ireland Galway, School of Nursing and Midwifery, University Road, Galway, Ireland; 6 University of Ottawa, Institute of Population Health, 1 Stewart Street, Ottawa, ON, K1N 6N5, Canada

**Keywords:** Core outcome set, Outcome reporting bias, Clinical trials, Systematic review, Methodology, Consensus

## Abstract

The selection of appropriate outcomes or domains is crucial when designing clinical trials in order to compare directly the effects of different interventions in ways that minimize bias. If the findings are to influence policy and practice then the chosen outcomes need to be relevant and important to key stakeholders including patients and the public, health care professionals and others making decisions about health care. There is a growing recognition that insufficient attention has been paid to the outcomes measured in clinical trials. These issues could be addressed through the development and use of an agreed standardized collection of outcomes, known as a core outcome set, which should be measured and reported, as a minimum, in all trials for a specific clinical area. Accumulating work in this area has identified the need for general guidance on the development of core outcome sets. Key issues to consider in the development of a core outcome set include its scope, the stakeholder groups to involve, choice of consensus method and the achievement of a consensus.

## Background

Clinical trials seek to evaluate whether an intervention is effective. This is determined by comparing outcomes that are chosen to reflect beneficial and harmful effects. Outcomes may be specific (for example, progression of loss of eyesight in patients with retinal degeneration) or they may be a broad construct used to demonstrate effects of an intervention on one aspect of health (for example, pain). Selection of appropriate outcomes or domains is crucial when designing clinical trials to compare directly the effects of different interventions in ways that minimize bias. If the findings are to influence policy and practice then the chosen outcomes need to be relevant and important to key stakeholders including patients and the public, health care professionals and others making decisions about health care.

There is a growing recognition that insufficient attention has been paid to the outcomes measured in clinical trials. Difficulties caused by heterogeneity in outcome measurement are well-known to systematic reviewers. For example, the most accessed and the top cited Cochrane Reviews in 2009 [1] all describe problems due to inconsistencies in the outcomes reported in trials. Furthermore, empirical research provides strong evidence that outcome reporting bias, defined as the results-based selection for publication of a subset of the original measured outcome variables, is an important problem in randomized trials [2] that affects the conclusions in a substantial proportion of Cochrane reviews [3]. Outcome reporting bias is likely to affect systematic reviews more widely, as well as published research in general.

These issues could be addressed through the development and use of an agreed standardized collection of outcomes, known as a core outcome set (COS), which should be measured and reported in all trials for a specific clinical area [4]. These sets do not imply that outcomes in a particular trial should be restricted to those in the COS. Rather, there is an expectation that the core outcomes will always be collected and reported, and that researchers will continue to explore other outcomes. In most trials, the primary outcome would be expected to be one of those contained in the COS. If a COS is not implemented in a particular trial, the researchers should explain their decision in the trial protocol and subsequent report. Similarly, if the primary outcome for a particular trial is not within the COS, then the relevance and importance of the chosen outcome should be explained. This approach would enhance the value of evidence synthesis by reducing heterogeneity in reported outcomes between trials and reducing the risk of outcome reporting bias, since trial reports will always include presentation of their findings for the core outcomes, as a minimum. Statistical power would be increased because fewer studies would have to be omitted from meta-analyses. The GRADE (Grading of Recommendations Assessment, Development and Evaluation) group (http://www.gradeworkinggroup.org) recognize the need to identify a relevant set of core outcomes, and recommends that up to seven patient-important outcomes are listed in the ‘Summary of Findings’ tables in systematic reviews. This is supported by Cochrane Reviews of the effects of healthcare interventions [5,6] and by the World Health Organization (WHO) in developing guideline recommendations [7].

An important rationale for COS is that outcomes reported for trials may not reflect endpoints that are meaningful for health service users. Examples exist where patients identified an outcome important to them as a group that might not have been considered by practitioners on their own [8-12]. Recognition of the importance of incorporating health service user opinion in COS development is increasing, but involvement has been limited to date.

The most notable work relating to outcome standardization has been conducted by the OMERACT (Outcome Measures for Rheumatology Clinical Trials) collaboration since 1992, which advocates the use of COS in clinical trials in rheumatology [13]. More than 50 other groups have been working on COS in specific areas of health care, including pain [14], maternity care [15] and some pediatric specialties [16].

The COMET (Core Outcome Measures in Effectiveness Trials) Initiative [17] brings together researchers interested in the development, application and promotion of COS, derived using rigorous consensus methods, for effectiveness trials. COMET aims to collate and stimulate the development of relevant resources, both applied and methodological, to facilitate exchange of ideas and information, to work with patients, the public and their representatives to develop material to improve health service user engagement, and to foster methodological research in the area of COS. Data on relevant individual studies, both published and ongoing, are being included in a free, publically available internet-based resource. This is a unique resource, which is updated periodically, and which should serve to minimize duplication of effort in the development of COS.

As each COS is developed, it will be important to agree on an appropriate instrument or definition for each included outcome. Examples of lack of validated instruments and standardized definitions abound in the literature. For example, fewer than 20% of 906 different outcomes measured in breast reconstruction surgery trials were defined or measured with a validated tool [18,19].

Accumulating work in this area has identified the need for general guidance on the development of COS. We identify here key issues to consider and methodological decisions to be made, referencing illustrative examples. Study protocols, in which key decisions are documented regarding the choices made in the process of COS development, are emerging, for example in the MOMENT study (The management of otitis media with effusion in children with cleft palate: a feasibility study http://www.hta.ac.uk/project/2555.asp) and the development of COS for surgery for colorectal and esophageal cancer, morbid obesity and breast reconstruction.

## Main text

### Suggested approach for development of a core outcome set

#### *Scope*

The specific area of health or healthcare to which the COS is to apply needs to be described, with details of health condition, population and types of interventions. The COS may be developed to encompass all stages or severity of a health condition or it may be focused on a particular disease category. For example, in colorectal cancer, a COS may be developed for all patients or it may focus on patients with metastatic disease. Similarly, the core set may be developed for all treatment types or for a particular intervention (for example, COS may be created for use in all trials of interventions to treat morbid obesity or for bariatric surgery alone).

#### *Identifying existing knowledge*

One of the difficulties in this area of research is how to identify studies that have already been done (or are underway) to develop COS. As part of the COMET Initiative, a searchable database has been developed [17]. This enables researchers to check for existing or ongoing work before embarking on a new project, thus minimizing duplication of effort.

A review of previous trials [20] or systematic reviews in the area can provide evidence of need for a COS and also identify a potential list of outcomes. Systematic reviewers are starting to use the outcome matrix recommended by the ORBIT project [3] to display the outcomes reported in the eligible studies. This matrix may demonstrate the inconsistency of outcomes measured to date in addition to potential outcome reporting bias. A review of studies other than clinical trials (for example, observational research into harms) may also identify additional outcomes, such as rare endpoints, that would be worthy of consideration for inclusion in the COS.

#### *Stakeholder involvement*

Key stakeholders may include patients and the public, health care practitioners, regulators, industry representatives, and researchers. Bringing diverse stakeholders together to try to reach a consensus is increasingly well-accepted as the future of collaborative, influential research. An important example of this is the work of the James Lind Alliance in determining important questions about treatments where uncertainty remains [21]. Decisions regarding the stakeholder groups to be involved and the target number from each group will be dependent upon the particular scope of the COS as well as upon existing knowledge and practical feasibility considerations. These decisions should be documented and explained in the study protocol. Consideration should be given to potential conflicts of interest within the group developing the COS (for example, the developers of measurement instruments in the area of interest or those whose work is focused on a specific outcome) [22].

Few COS studies to date have involved patients or the public, yet those that have done so have identified outcomes that were not previously identified by the other stakeholders [4,5,23-26]. Methods for identifying patients or their representatives include clinics, patient societies, advocacy groups, and care giver support groups. To achieve representativeness, it may be helpful to consider approaching practitioners with the support of professional bodies.

#### *Consensus methods*

The first step in the process is typically to develop consensus about ‘what’ to measure. The ‘how’ and ‘when’ to measure are usually later in the process but may be determined by consensus methods as well. Groups that first establish consensus about what concepts to measure may subsequently conclude that there is a single measurement instrument for an outcome in the COS that is supported by sufficient evidence to recommend its use. However they may identify that gaps in outcome measurement exist, either because there is no ideal instrument for a particular concept or because the evidence base for existing instruments is of limited quality. These limitations may make it difficult to identify which of several potential measures may be preferable for use in the relevant context. The COSMIN (COnsensus-based Standards for the selection of health Measurement Instruments, http://www.cosmin.nl) checklist [27] can be used as a tool for developing studies of the validity and reliability of measurement instruments because it describes the necessary design requirements for the assessment of those measurement properties. In addition, feasibility of measurement is a further consideration [13].

Methods used in previous studies to elicit opinions and to develop consensus about important outcomes include expert panel meetings [28], Delphi surveys [22], Nominal Group Techniques [13], focus groups [24], individual interviews [25] and individual questionnaires [5]. Considerations concerning the choice of method include the need to build a true consensus with methodological rigor, strategies to ensure that a diverse range of opinions are heard, and factors such as financial and carbon costs that might limit the practicality of face-to-face meetings. It is important to ensure that views from all key stakeholder groups are included when making the final decision regarding the COS. This can be achieved through anonymous voting facilitated through email or keypads at meetings.

It is necessary to decide what information about possible outcomes should be given to stakeholder participants before a consensus exercise begins. A literature review of relevant studies showing the outcomes clinicians value and report most frequently, which may include information about methods to measure the identified outcomes and when to measure them, has been proposed [18-20]. This should be combined with outcomes deemed to be important to health service users if such work has been undertaken previously. If consensus participants are shown a list of potential outcomes, it is generally recommended that they be given the opportunity to propose the inclusion of additional items, especially as the literature may not include outcomes associated with the most recent treatments available. Because this has the potential to result in a long list of items, criteria for determining inclusion of items to be considered in the consensus exercise may be needed. For example, Devane *et al.*[15] required new items to have been suggested by at least two participants. If it is felt that the sharing of a list of outcomes at the outset of the consensus process may bias responses, open questioning may be preferred. Techniques to do this include administering questionnaires [5], focus groups [24] and in-depth interviews [25], to determine outcomes important to patients. However this may lead to stakeholders not considering areas previously deemed important, and subsequent questions to prompt consideration of specific outcomes may be warranted.

Researchers should consider the potential impact of the following methodological decisions on the final results: group composition, questioning technique, the information participants receive to inform their answers, whether or not responses are anonymous, how the group participants interacted with or influenced each other, the medium of the interaction, attrition bias, analysis which can miss or overstate the importance of certain outcomes, and the way in which consensus is reached. A single heterogeneous consensus panel comprising the various stakeholders may be deemed appropriate for particular areas of healthcare whereas separate panels for different stakeholder groups followed by work to integrate the multiple perspectives may be more appropriate for others. A variety of methods have been used to date in published and ongoing studies, particularly related to the inclusion of the patient perspective. The proportion of patients and health service users chosen in a Delphi survey may depend upon the clinical setting; for example, for breast reconstructive surgery, which is an optional procedure undertaken for cosmetic purposes, the involvement of patients is more critical than in other settings. A research project has recently started within the COMET Initiative to assess the effectiveness of these different methods.

Consideration should be given in advance to the criteria that will be used to determine when consensus has been achieved. A review of the reporting of Delphi studies to develop COS demonstrated poor reporting of the methods used [29]. As an example definition, in a Delphi survey participants may be asked to score each outcome from a long list using the scale proposed by the GRADE group http://www.gradeworkinggroup.org, in which 1 to 3 signifies an outcome of limited importance, 4 to 6 important but not critical, and 7 to 9 critical. A number of rounds may be held in which responses are summarized and fed back to individuals, allowing them to change their score in light of the group’s opinion. Consensus regarding whether an outcome should be in the COS could be defined as 70% or more of the respondents scoring it 7 to 9 and fewer than 15% scoring it as 1 to 3. Consensus that an outcome should not be included in the COS could, for example, be defined as 70% or more scoring it as 1 to 3 and fewer than 15% scoring it as 7 to 9. All other score distributions would be taken to indicate lack of agreement for inclusion of a given outcome in the COS. The rationale for these thresholds is that acceptance that consensus has been reached for an outcome to be included in the COS requires agreement by the majority regarding the critical importance of the outcome, with only a small minority considering it to have little or no importance. Likewise, for consensus to have been reached that an outcome should not be in the COS requires agreement by the majority that the outcome is of little or no importance, with only a small minority considering it to be critically important. Whereas choice of thresholds is somewhat subjective, specification of the definition in the study protocol should reduce the risk that researchers will define consensus post-hoc in a way that would bias the conclusions toward their own beliefs.

Consideration should be given in advance to the possibility that consensus may not be achieved. For example, different stakeholders may disagree about the inclusion of specific outcomes in the COS. This might lead to a decision to recommend the smaller COS, about which there is consensus.

#### *Achieve global consensus*

To compare and contrast all research in a topic area, a COS must be applicable and adopted across international settings and across relevant disciplines. Expert panels and conference workshops have been used to achieve international consensus [13].

#### *Review with feedback and updating as necessary*

Opportunities to review the COS periodically are important as a form of validation, to ensure outcomes are still relevant and important, to allow the chance to add new outcomes, to evaluate how successful implementation has been and to engage further stakeholders as appropriate. The question of who should be developing and reviewing COS (for example, professional organizations or groups of trialists in particular therapeutic areas) needs careful consideration to ensure continued support for the activity.

#### *Implementation of core outcome set*

To increase COS uptake, it is recommended that developers consider engagement with the relevant Cochrane Review Groups, clinical guideline developers, research funders, journal editors, regulators such as research ethics committees, and trial registries. For example, the NIHR Health Technology Assessment funding body in the UK has recently added the following statement to its application form:

"‘Details should include justification of the use of outcome measures where a legitimate choice exists between alternatives."

"- Where established Core Outcomes exist they should be included amongst the list of outcomes unless there is good reason to do otherwise. Please see The COMET Initiative website at http://www.comet-initiative.org to identify whether Core Outcomes have been established.’"

Potential barriers and cost implications of implementing the COS should be considered.

#### *Clear and transparent presentation*

Reporting standards for studies specifically using Delphi methods to achieve consensus about a COS have been recommended [29]. We propose the checklist in Table 1 to improve the reporting quality of studies to develop consensus around domain concepts or what to measure more generally.

**Table 1:** Checklist of the items that groups should consider when reporting the development of a COS of domain concepts (that is, ‘what’ to measure)

**Section/topic**	**#**	**Checklist item**
Title	1	Identify the report as a study to develop a COS.
Structured summary	2	Provide a structured summary including, as applicable: background, objectives, data sources, participant eligibility criteria, study methods, results, limitations, conclusions, and implications of key findings.
Rationale	3	Describe the rationale for the development of a COS in the context of what is already known. This may include a review of outcomes in previous trials or systematic reviews.
Objectives	4	Provide an explicit statement of questions being addressed with reference, as applicable, to: health condition, population, and types of intervention(s).
Protocol and registration	5	Indicate if a study protocol exists, and where it can be accessed (for example, web address)
Eligibility criteria	6	Specify participant eligibility criteria, including stakeholder group, the rationale for involving them, and how participants were identified and sampled.
Information sources	7	Describe all information sources (for example, systematic review, databases with dates of coverage, contact with study authors) provided to participants before the start of and during the consensus process. If no information on previously measured outcomes is provided, this should be clearly stated together with details of the method for obtaining information on outcomes of importance from the participants.
Consensus process	8	Describe method to determine consensus and the rationale. A checklist for reporting Delphi methods applied to the development of COS has previously been recommended [29].
Outcome scoring	9	Describe how outcomes will be scored during the consensus exercise, and how scores will be summarized across participants during each stage of the consensus process.
Definition of consensus	10	Clearly describe any pre-determined definition of consensus. Describe procedure for determining how outcomes will be included or excluded from consideration at each stage of the consensus process.
Participants	11	Give the total number of participants invited and the number involved in each aspect of the study. Give the proportion of each type of participant from the various stakeholder groups involved. Present any data collected on participant characteristics.
Results of the consensus process	12	As a minimum, provide a comprehensive list of all the outcomes that participants agreed should be included in the core set. Describe a measure of group response and distribution of response for each outcome considered during the process.
Summary of evidence	13	Summarize the main findings regarding the level of consensus and the content of the COS. Consider its relevance to key groups e.g. patients and the public, healthcare providers, and policy makers, and any potential barriers to implementation.
Limitations	14	Discuss limitations in terms of stakeholder and geographical coverage. Describe methods used for assessing risk of bias, in relation to information provided to participants beforehand, attrition, any lack of anonymity, etc.
Conclusions	15	Provide a general interpretation of the results in the context of other evidence, and implications for future research.
Funding	16	Describe sources of funding and the role of the funder in the study.
Conflicts of interest	17	Describe any conflicts of interest within the study team, for example researchers who have developed an outcome measurement instrument applicable to the scope of the COS.

This includes declaration of potential conflicts of interests within the COS development study team to avoid concerns that individuals with vested interests; for example, the developers of measurement instruments in the area of interest may have overtly or covertly manipulated the process of consensus development.

### Registration with COMET

One of the aims of the COMET Initiative is to provide a means of identifying existing, ongoing and planned COS studies. COS developers should be encouraged to register their project with COMET. The following information about the scope and methods used will be recorded in the database for existing and ongoing work:

1. Clinical areas for which the outcome domains or outcomes are being considered, identifying both primary disease and types of intervention.

2. Method of development to be used for the COS, including the information participants will receive beforehand to inform their answers, and the methods to be used to reduce any list of possible outcomes.

3. People and organizations involved in identifying and selecting the outcome domains or outcomes, recording how the relative contributions will be used to define the COS.

4. The geographical setting(s) of the study.

Details of any associated publications, including the final report describing whether consensus has been reached, and the outcome domains and outcomes that were included in the COS, will be recorded in the database. Currently, the database includes studies ranging from the development of a specific COS using formal consensus methods, to a systematic review of outcomes measured in clinical trials in a particular area that may inform future COS development. Articles are classified according to the methods used therein.

## Discussion

Various methods have been used to develop a COS and it is uncertain which are most suitable, accurate and efficient. There is limited empirical evidence regarding whether different methods lead to similar or different conclusions. One example where consensus work has been undertaken in two different ways is in pediatric asthma. The American Thoracic Society/European Respiratory Society employed an expert panel approach [28], whereas other researchers combined results from a Delphi survey with clinicians and interviews with parents and children. The results were overlapping but not identical [10].

A systematic review of all COS development projects is ongoing, extending a previous review of pediatric studies [16]. This involves a survey of and interviews with COS developers in order to understand their choice of approach, to describe their experience of COS development and implementation, and to identify priority areas for methodological research. It is evident that the reporting of COS development studies requires improvement [29], and as the number of COS studies increases it is important to identify a minimum amount of information to be included in future publications. It is to be expected that the proposed checklist (Table 1) will develop over time, in a similar way to the CONSORT statement [30], based on accumulating evidence. A quality assessment tool will also be developed, using accepted methods [31].

The methods for COS development need to be sufficiently rigorous so as to avoid those situations in which a COS is recommended but subsequently found to be deficient in some way because of the methods used to develop it. Establishing a COS using rigorous methods will also help in setting the threshold for changing it, which arguably should be high, as it is in OMERACT [13]. For example, the original OMERACT core set in rheumatoid arthritis was established without direct patient input; but focus groups were held at OMERACT 6 (in 2002), the first OMERACT meeting that patients were invited to attend. Supported by a previous email survey, fatigue and sleep were identified as missing from the OMERACT core set, which only included pain, function, joint counts, global assessments and a blood test. The experience of fatigue has been reported by a large proportion of people with rheumatoid arthritis, and it is often the most important problem for individual patients. A systematic analysis of these patient focus group discussions revealed three overarching themes: fatigue is overwhelming and different from normal tiredness; it permeates every sphere of life; and self-management is variable, but professional support is rare. Over the next two years a systematic search for articles measuring fatigue discovered twenty-three scales, six of which had sufficient evidence of validity to pass most of the OMERACT filter for truth, discrimination and feasibility [13]. Further work to demonstrate responsiveness was then undertaken and in 2006 fatigue was endorsed as an additional core outcome by a vote at the OMERACT 8 meeting [32].

As noted above, another important consideration is the question of who should be developing and reviewing COS (for example, professional organizations or groups of trialists in particular therapeutic areas). The collation and review of empirical evidence concerning dissemination methods and strategies to encourage uptake are other important aspects of the work that need to be considered early in the process.

## Conclusion

The COMET Initiative provides a focus for the continued development of a framework for outcome measurement, first in relation to domains and outcomes within domains, subsequently in terms of definitions and measurement instruments, and finally in relation to the timing of measurement. There is an increasing awareness of the need for greater attention to be given to the outcomes measured in clinical trials, in terms of standardization and reporting. Consideration of the issues and the checklist described should help with the development, reporting and implementation of COS.

## Abbreviations

COMET: Core Outcome Measures in Effectiveness Trials; CONSORT: CONsolidated Standards Of Reporting Trials; COS: core outcome set; COSMIN: COnsensus-based Standards for the selection of health Measurement INstruments; GRADE: Grading of Recommendations Assessment: Development and Evaluation; MOMENT: The management of otitis media with effusion (OME) in children with cleft palate: a feasibility study; NIHR: National Institute for Health Research; ORBIT: Outcome Reporting Bias in Trials; OMERACT: Outcome MEasures for Rheumatology Clinical Trials; WHO: World Health Organization.

## Competing interests

The authors declare that they have no competing interests.

## Authors’ contributions

PRW conceived of the article, and wrote the first draft of the manuscript. DGA helped to revise the draft manuscript. JMB helped to revise the draft manuscript. MC helped to revise the draft manuscript. DD helped to revise the draft manuscript. EG helped to revise the draft manuscript. PT helped to revise the draft manuscript. All authors read and approved the final manuscript.
